# Combining Topography and Chemistry toward Polydopamine Antibacterial Surfaces

**DOI:** 10.1021/acsbiomedchemau.5c00242

**Published:** 2026-04-19

**Authors:** Leonardo Moscolari, Simona Tomaselli, Francesco Galeotti, Erika Kozma

**Affiliations:** Istituto di Scienze e Tecnologie Chimiche “G. Natta” (SCITEC), Consiglio Nazionale delle Ricerche, via A. Corti 12, Milano 20133, Italy

**Keywords:** polydopamine, cellulose acetate butyrate (CAB), silver nanoparticles, patterned structures, surface chemistry, antibacterial

## Abstract

Microporous antibacterial surfaces offer a promising route for preventing bacterial adhesion and biofilm formation, yet their fabrication often involves complex and energy consuming processes. In this work, we present a sustainable strategy for producing multifunctional antibacterial surfaces by combining a microporous cellulose acetate butyrate (CAB) substrate, a bioinspired polydopamine (PDA) coating, and in situ synthesized silver nanoparticles (AgNPs). CAB, selected for its film-forming capability and ease of microstructuring via spin-coating, provides a tunable platform for topographical control. PDA, formed through the oxidative self-polymerization of dopamine, serves as a conformal adhesive layer and simultaneously acts as a reductive matrix for AgNP formation. Immersion in silver nitrate solution enables the direct deposition of AgNPs without external reducing agents, facilitated by the catechol functionalities of PDA. The resulting CAB–PDA–AgNP surfaces exhibit enhanced antibacterial activity through the combined effects of patterning, surface chemistry, and silver-mediated bactericidal action. The fabrication process is low-energy and solvent-minimized, aligning with principles of sustainable material development and offering a versatile platform for antibacterial coatings.

## Graphical abstract

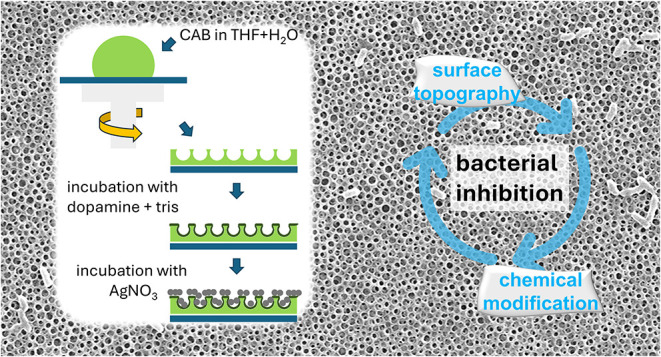

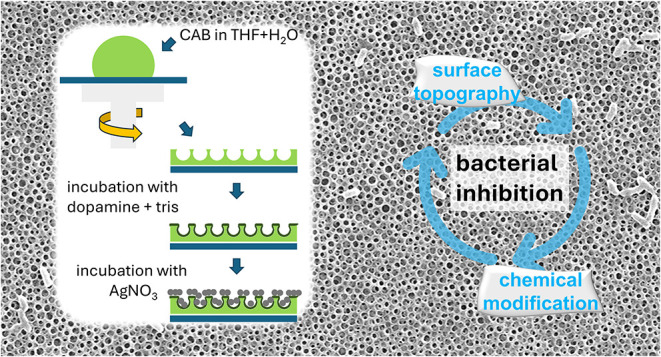

## 1 Introduction

Bacterial contamination of surfaces remains a significant challenge across diverse sectors, including healthcare, food packaging, and public infrastructure,^1^ driving the growing interest in antibacterial surfaces as a proactive strategy to prevent bacterial colonization and biofilm formation, thereby reducing infection transmission and enhancing public health safety.^2^ Antibacterial surfaces, increasingly explored as a strategy to mitigate bacterial contamination, are divided into passive and active types according to their underlying mechanisms of action.^3^ Passive antibacterial surfaces are designed to prevent bacterial adhesion by modifying the physicochemical properties of the material surface, employing strategies such as tuning surface topography, wettability, and surface charge to inhibit bacterial adhesion, growth, and proliferation. For instance, micro- and nanopatterned surfaces can physically deter bacterial colonization by creating topographies that are unfavorable for adhesion or that mechanically disrupt bacterial membranes.^4–6^ Similarly, superhydrophilic or superhydrophobic surfaces can reduce bacterial attachment by minimizing the contact area between the bacteria and the substrate.^7^

In contrast, active antibacterial surfaces incorporate agents that directly kill or inhibit bacteria. These agents can be either embedded within the material or immobilized on its surface and include metal ions (e.g., Ag^+^, Cu^2+^, and Zn^2+^),^8^ antimicrobial peptides,^9^ and nanozymes.^10^ The latter exhibits catalytic activity that generates reactive oxygen species or disrupts bacterial metabolism. Active agents may act upon direct contact or be released in a controlled manner to maintain a long-term antibacterial efficacy. The effectiveness of these surfaces depends not only on the nature of the agent but also on its stability, release kinetics, and interaction with the surrounding environment.

More recently, hybrid and stimuli-responsive antibacterial surfaces (smart surfaces) have been developed to combine the benefits of both passive and active mechanisms.^11^ These advanced materials can respond to external stimuli such as pH, temperature, light, or bacterial enzymes, triggering the activation or release of antibacterial agents only when needed. This targeted approach enhances specificity, reduces unnecessary exposure to antimicrobials, and helps mitigate the development of resistance.

These strategies are part of a growing effort to design surfaces that are not only effective in preventing bacterial contamination but also biocompatible, durable, and resistant to biofouling over time.

In this work, we present a sustainable and modular approach for engineering multifunctional antibacterial surfaces. Our strategy is based on the integration of three key components: a microporous cellulose acetate butyrate (CAB) substrate, a bioinspired polydopamine (PDA) coating, and a green method for the in situ synthesis of silver nanoparticles (AgNPs).

CAB was selected as the substrate material due to a combination of practical and performance-related advantages. As a chemically modified natural polymer, it is widely used across industries—from automotive coatings and packaging to cosmetics and pharmaceuticals—making it both accessible and cost-effective.^12^ Its tailored acetylation and butyration processes enhance solubility in various organic solvents, which is particularly beneficial for techniques like spin-coating.^13^ Additionally, the degree of substitution in CAB can be precisely controlled, allowing customization of its physical and chemical characteristics. Its excellent film-forming ability ensures the production of stable layers. Furthermore, CAB offers desirable mechanical traits, such as flexibility and toughness, which are crucial for preserving the structural integrity of porous scaffolds throughout fabrication and subsequent use. However, studies exploring the patterning of CAB remain relatively limited, which further motivated its selection in this study.^14–17^

We employed the breath figure (BF) method to fabricate microporous CAB surfaces.^18,19^ This technique provides a straightforward and economical route to highly ordered porous morphologies, overcoming limitations of traditional approaches that rely on cleanroom-based photolithography, micromolding from lithographically produced masters, or plasma micromachining. It involves casting or spin-coating a polymer solution under humid conditions, where solvent evaporation induces condensation of water droplets on the surface. These droplets act as dynamic templates for pore formation; upon their evaporation, they leave behind a regular array of pores that replicate the droplet pattern. The water required for droplet formation during the BF process can originate either from a controlled stream of humid air directed over the polymer solution or from water intentionally added to the solution itself.

PDA has emerged as a remarkably versatile material in the field of biomedical engineering, inspired by adhesive proteins secreted by mussels. These marine organisms adhere to virtually any surface under wet conditions, a property attributed to the catechol and amine functionalities in their proteins. Mimicking this natural mechanism, PDA is formed through the oxidative self-polymerization of dopamine in mildly alkaline conditions, resulting in a conformal, hydrophilic coating that adheres strongly to a wide range of materials, from metals and ceramics to polymers. PDA not only provides a robust adhesive layer but also offers reactive sites for further chemical modification, including the immobilization of biomolecules, drugs, or nanoparticles.^20^

Although PDA has been widely studied for its adhesive and surface-modifying properties, its antibacterial activity is supported by a relatively limited number of studies. One proposed mechanism involves a contact-active process, in which catechol moieties and protonated amine groups interact with bacterial membranes, leading to protein denaturation, membrane disruption, and ultimately cell lysis, similar to the effects observed with plant-derived polyphenols.^21^ This mechanism has been suggested by findings such as those reported by Karkhanechi et al.,^22^ who attributed PDA’s biofouling resistance to these functional groups. Additionally, PDA’s antimicrobial properties have been linked to the generation of reactive oxygen species via electron transfer processes^23^ and to a contact-mediated bactericidal effect influenced by the physicochemical characteristics of the PDA layer. The latter is closely associated with variations in surface morphology—specifically thickness and roughness—arising from distinct polymerization parameters, including dynamic versus static deposition conditions and substrate orientation during film formation (horizontal vs vertical).^24,25^

Taking advantage of the intrinsic reductive properties of PDA, we achieved the in situ synthesis of AgNPs on CAB–PDA films via a straightforward immersion in a silver nitrate solution. The catechol groups in PDA efficiently reduced Ag^+^ ions to metallic silver, eliminating the need for external reducing agents or stabilizers, while simultaneously promoting strong adhesion of the AgNPs to the substrate surface. This dual functionality of PDA, as both a reducing agent and an adhesive matrix, was previously demonstrated by Liu et al., who successfully employed it to fabricate stable multilayer AgNP coatings on contact lens surfaces.^26^ Moreover, the in situ formation of various metal nanoparticles can impart additional functionalities such as catalytic activity or plasmonic amplification of Raman signals in sensing applications.^27–29^ Notably, this PDA-mediated reduction route bypasses several steps commonly required in conventional nanoparticle deposition procedures, such as the use of external chemical reducing agents, high-temperature treatments, or additional stabilizing ligands, allowing metal NP formation to proceed under mild ambient conditions.

This integrated design not only conveys strong antibacterial activity through the synergistic effects of microstructuring, surface chemistry, and AgNP deposition but also establishes a low-energy, solvent-minimized fabrication process aligned with sustainable material development.

## 2 Materials and Methods

### 2.1 Materials

Cellulose acetate butyrate (CAB), tri­(hydroxymethyl)­aminomethane (tris)-HCl, silver nitrate (AgNO_3_), and tetrahydrofuran (THF) were purchased from Merch Chemicals, and dopamine hydrochloride was purchased from Fluorochem. All chemicals were used as received, without further purification.

*Escherichia coli* (ATCC 11229) and *Staphylococcus aureus* (ATCC 6538) were purchased from Biogenetics Diagnostics. The staining agent SYTO9 was purchased from Molecular Probes, USA.

### 2.2 Preparation of CAB Films

CAB was dissolved in tetrahydrofuran (THF) at a concentration of 50 g/L. To prepare the porous films, 5% (v/v) Milli-Q water was added to the THF solution (optimized condition) and 50 μL of this mixture was deposited on a clean round coverslip (Ø = 12 mm) and spin-coated under ambient conditions at 2000 rpm (P2000) and 4000 rpm (P4000). To prepare the flat films, CAB solution in dry THF was deposited on a clean round coverslip (Ø = 12 mm) and spin-coated at 3000 rpm. To avoid any introduction of humidity, the spin-coating process was performed in a dry glovebox.

### 2.3 Polydopamine Coating

In a Petri dish (Ø = 90 mm), 80 mg of dopamine hydrochloride was dissolved in 40 mL of a 25 mM tris solution (pH = 9). To avoid delamination from the border due to surface tension, the CAB samples (flat, P2000, and P4000) were protected with an external poly­(dimethylsiloxane) ring. Furthermore, just before immersion, a drop of ethanol was placed on the porous samples to increase hydrophilicity and immediately immersed in the dopamine solution for 2 h under stirring at room temperature. Then, the samples were rinsed with water and incubated again in fresh dopamine solution for another 2 h. Finally, the PDA-coated samples were rinsed with water and dried at room temperature. The obtained samples are FLAT PDA (polydopamine deposited on flat CAB), P2000-PDA (PDA deposited on microporous CAB spin-coated at 2000 rpm), and P4000-PDA (PDA deposited on microporous CAB spin-coated at 4000 rpm).

### 2.4 Silver Nanoparticle In Situ Growth

The PDA-coated samples were placed on the bottom of a 50 mL crystallizer in a 4 g/L water solution of AgNO_3_. The reaction solution was stirred gently for 4 h. Samples were rinsed with water and dried at room temperature.

### 2.5 Viability Tests for Antibacterial Activity Detection

Bacteria were recovered from −80 °C stocks in rich media (rm): peptone water/yeast agar, purchased from Merck. After overnight (o.n.) growth, bacterial cultures were diluted 1:5000 in a minimal medium (m.m.), a modified M9 medium adapted for experimental needs, containing 0.02 M NaCl, 0.02 M NH_4_Cl, 0.022 M KH_2_PO_4_, 0.048 M Na_2_HPO_4_, 2.0 mM MgSO_4_, 0.1 mM ZnSO_4_, 0.2 mM FeCl_3_, and 0.8% glucose. Volumes of 80 μL of the preinoculated minimal medium (m.m.) were deposited onto all tested materials and incubated overnight at 37 °C under static conditions. The culture (10 μL) was then recovered, diluted, plated, and grown overnight at 37 °C. Real surface areas were estimated from SEM micrographs by extracting pore size and depth and modeling each pore as a truncated conical cavity. The developed surface area was obtained from the lateral area of the pores multiplied by their packing density, and the result was scaled to the full 10 mm diameter sample area. Planktonic cells are defined as bacteria remaining in suspension in the culture medium after 24 h of incubation on the tested materials. Planktonic viability was quantified by plating appropriate dilutions of the supernatant: 1 mL of diluted samples was plated on an agar medium, and colony-forming units (CFUs) were counted after overnight incubation at 37 °C. Sessile cells (biofilm-forming cells) are defined operationally as surface-attached bacteria that remain on the material after two (1 mL each) gentle washes in 0.9% NaCl and are subsequently detached (in 1 mL of fresh 0.9% NaCl solution) by 15 min of sonication for viable counting. Aliquots of 1 mL were then appropriately diluted, plated on agar, and incubated overnight at 37 °C, and CFUs were counted to assess sessile viability after sonication. The green fluorescent nucleic acid staining agent SYTO9 was used to label the adherent bacterial cells on the samples. The samples with adherent bacteria were washed two times with NaCl 0.9% and incubated with 100 μL of solution containing SYTO9 (3.34 mM) at room temperature in the dark for 20 min. Viability data were plotted as CFU/cm^2^ and were normalized by the exposed sample area. The limit of detection (LOD) of the plating assay was calculated based on the minimum detectable colony count (1 CFU), the dilution factor, the recovered sample volume, and the sampled surface area. The LOD in CFU/cm^2^ was determined according to the following equation:

$${\mathrm{LOD}}_{\mathrm{CFU}/{\mathrm{cm}}^{2}}=(N_{\min}\;\times \;\mathrm{DF}\;\times \;V_{\mathrm{rec}})/A$$

where *N*_min_ is the minimum detectable number of colonies on the agar plate (1 CFU), DF is the dilution factor, *V*_rec_ is the recovered volume from the sampled surface (mL), and *A* is the sampled area (cm^2^).

In the present experimental setup, the following LODs were calculated (expressed as CFU/cm^2^): 12 for flat CAB, 10 for P2000, and 11 for P4000 samples. Values below this threshold were considered below the detection limit. Samples for SEM were prepared by depositing 80 μL of the preinoculated m.m. onto all tested materials, incubated overnight at 37 °C under static conditions, and washed with 0.9% NaCl.

### 2.6 Statistical Analysis

All viability data were analyzed using Sigmaplot software (Version 12.0 for Windows) and were expressed as the mean ± standard deviation of three independent experiments. Differences between samples were assessed by one-way analysis of variance (ANOVA) followed by the Bonferroni post hoc test. A 95% confidence level was applied to all statistical analyses, and*p*-values ≥0.05 were considered not statistically significant. Statistical analysis of flat CAB PDA-coated samples with respect to not coated flat CAB was evaluated using two-tailed Student’s *t* test.

### 2.7 Imaging and Surface Characterization

SEM analysis was performed by using a Phenom Pro Desktop scanning electron microscope (Thermo Fisher Scientific Inc., Eindhoven, The Netherlands), at an accelerating voltage of 15.0 kV, after sputtering the samples with 5 nm of Au. The samples containing bacteria were fixed before imaging in 0.9% NaCl solution containing 2.5% of glutaraldehyde at 4 °C overnight. Then, the samples were rinsed with 0.9% NaCl solution and further dehydrated using serial diluted ethanol solutions (30, 50, 70, 90, and 100%), for 10 min in each solution, and dried at room temperature. AFM investigations were performed using an NT-MDT NTEGRA apparatus in tapping mode under ambient conditions. The surface wettability of the substrates was investigated by using an Ossila (Leiden, NL) contact angle goniometer. The average contact angles were calculated from six different testing spots on each substrate.

## 3 Results and Discussion

The fabrication process of the engineered CAB/PDA/Ag surfaces is depicted in Figure 1.

**Figure 1:**
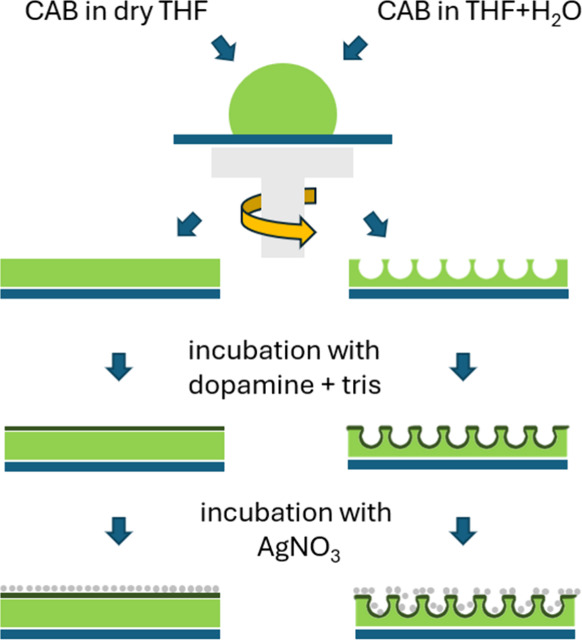
Schematic illustration of flat and microporous CAB films followed by surface functionalization with dopamine and electroless silver nanoparticle deposition.

In this study, spin-coating was employed to fabricate micropatterned polymer films by BFs due to its superior reproducibility and rapid processing compared with drop-casting techniques. To address the challenges associated with humidity control during BF formation, a ternary system was adopted by introducing controlled amounts of water into a CAB solution in THF. THF serves as the primary solvent for CAB, effectively dissolving CAB, while water, being miscible with THF but not a solvent for CAB, promotes phase separation and facilitates pore nucleation. Indeed, the incorporation of water modulates the evaporation dynamics and interfacial tension, enabling the formation of patterned structures. By variation of the water content, along with other process parameters, including polymer concentration, spin-coating speed (rpm), and other conditions, the pore size can be effectively tuned. These details are thoroughly described in our previous work.^30^

For the purposes of this study, we selected two different porosities, P2000 and P4000, corresponding to spin-coating speeds of 2000 and 4000 rpm, respectively.

Under these conditions, highly uniform surfaces were obtained, with average pore diameters of approximately 640 nm for P2000 and 470 nm for P4000 (Figure 2). As previously demonstrated, in fact, the average pore size decreases with increasing spin-coating speed, indicating an inverse correlation between spinning rate and pore dimension.^30^ Flat films were also prepared, for comparison, by spin-coating the CAB solution in dry THF. The thicknesses of flat and porous films, measured by profilometry, were 810 and 960 nm, respectively. The real surface areas, estimated from SEM images, were 0.785 cm^2^ for flat films, 0.943 cm^2^ for P2000, and 0.950 cm^2^ for P4000.

**Figure 2:**
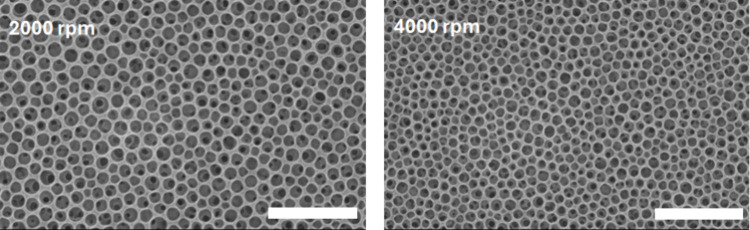
SEM micrographs of CAB films obtained by spin-coating at 2000 and 4000 rpm spinning rates. Scale bars are 5 μm.

To functionalize the flat and patterned CAB surface, a PDA coating was applied via the oxidative self-polymerization of dopamine under mildly basic conditions. This process enabled the formation of a uniform PDA coating. The thickness of the PDA layer, measured by AFM on flat samples, was 20 ± 5 nm, confirming the result already observed in our previous study.^31^ PDA deposition under these conditions preserves the underlying microstructure while imparting adhesive and redox-active functionalities essential for subsequent surface modifications. To prevent the PDA film from preferentially forming over the cavities of the CAB scaffold, potentially sealing them rather than preserving the porous morphology, we introduced a preconditioning step using ethanol. This treatment enhanced the wettability of the CAB surface, facilitating deeper infiltration of the dopamine solution into the air-filled pores and promoting a conformal coating along the internal scaffold architecture.

To further preserve the porous architecture imparted by CAB, we implemented a two-step incubation protocol designed to minimize the formation of PDA particles in solution, an issue commonly observed during prolonged reaction times. These particles tend to adhere to the PDA-coated surfaces, potentially altering their morphology. By adopting two steps of a 2 h incubation, separated by a water rinsing and a renewal of the dopamine solution, we effectively reduced unwanted particle deposition and maintained the integrity of the original porous structure.

Surface wettability is a critical parameter governing interfacial phenomena, particularly in applications involving biological interactions and adhesion. To assess the surface modifications induced by PDA coating, water contact angle (WCA) measurements were performed on both flat and microporous surfaces (Figure 3).

**Figure 3:**
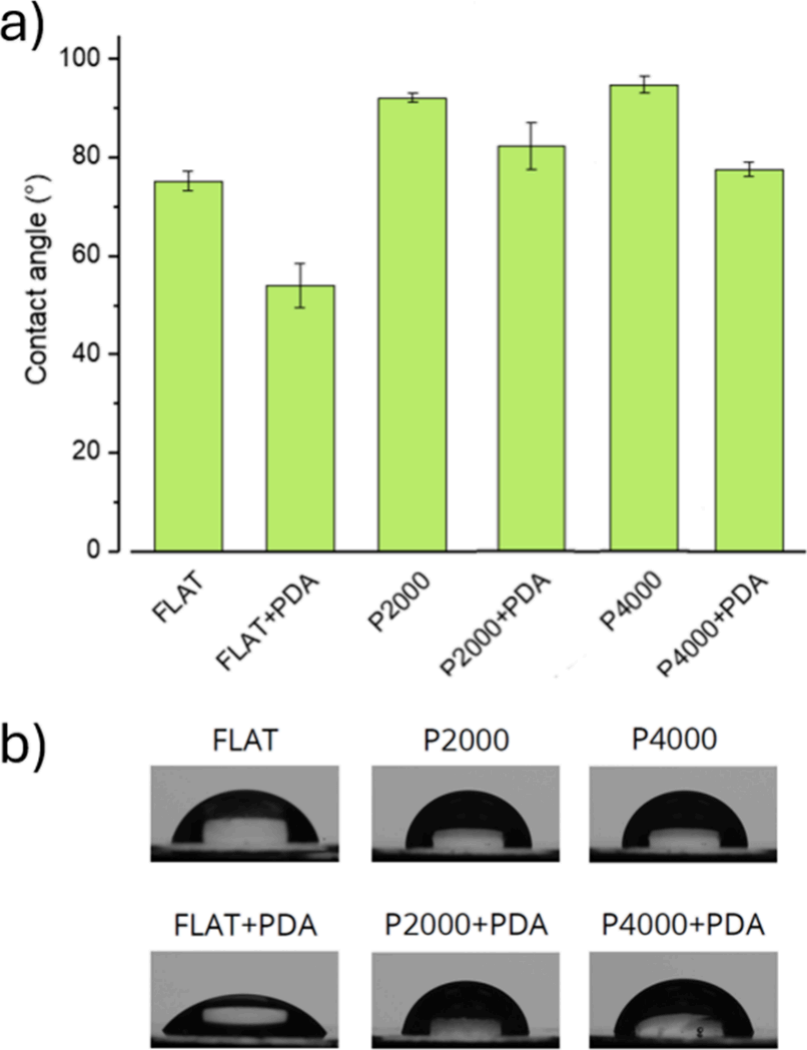
Water contact angle measurements for flat and microporous surfaces with and without PDA coating (a) and view of the water droplet shape deposited on the different samples (b).

The unmodified flat CAB film exhibited a WCA of 75°, indicating a moderately hydrophilic surface. Upon incorporation of PDA, the WCA decreased to 55°, demonstrating a significant enhancement in the surface hydrophilicity. The microporous CAB surfaces P2000 and P4000 showed a more hydrophobic behavior than the flat films, with WCAs of 91 and 93°, respectively, as expected in view of the increased surface roughness caused by their porous topography. However, after surface modification with PDA, both surfaces transitioned to a less hydrophobic state, as evidenced by a decrease of about 10% in their WCA. This transition to hydrophilicity across all samples after PDA coating is attributed to the presence of hydrophilic functional groups, including hydroxyl (−OH), amine (−NH_2_), and carboxyl (−COOH) moieties. These groups enhance the surface energy and promote stronger interactions with water molecules, thereby lowering the WCA and improving wettability.

The PDA-coated substrates were subjected to silver nitrate treatment under controlled conditions to facilitate the in situ formation of AgNPs. UV–vis spectroscopy confirmed the successful growth of AgNPs on the flat PDA-coated surfaces, as indicated by the emergence of the characteristic surface plasmon resonance (SPR) band for silver, centered from 418 to 428 nm.^32^ As shown in Figure 4a, the intensity of the SPR band can be modulated by adjusting both the AgNO_3_ concentration and the incubation time. The most pronounced signal was observed at 4 g/L after 4 h of incubation, indicating extensive nanoparticle growth and possibly the onset of aggregation, as suggested by the broadening of the spectral band. The consistent peak position across all samples implies that the AgNPs predominantly remain within the 20–60 nm size range, with minimal spectral shifts. These observations were corroborated by AFM analysis, which revealed a progressive increase in surface roughness and nanoparticle coverage, confirming the trend associated with longer incubation times and higher precursor concentrations.

**Figure 4:**
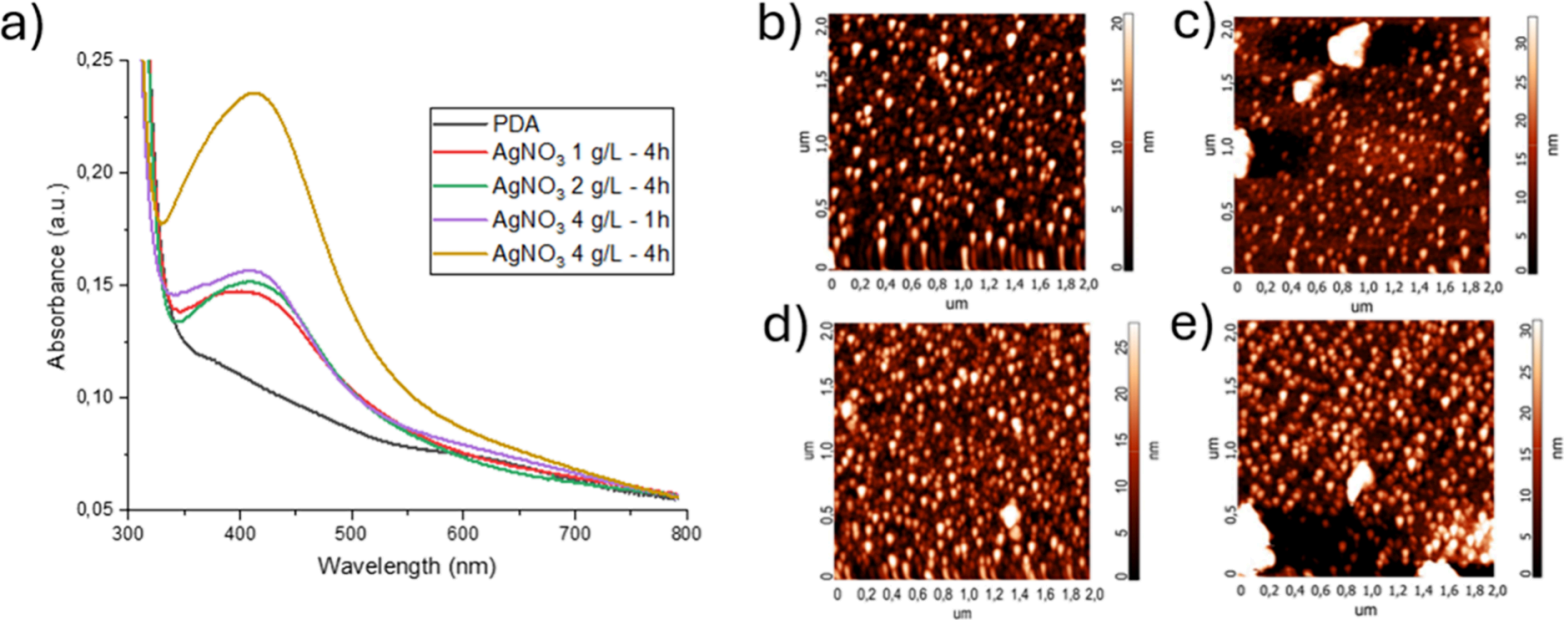
(a) UV–vis absorption curves of the PDA-coated flat CAB surface before and after incubation with AgNO_3_ in different conditions. (b–e) Corresponding 2.0 × 2.0 μm^2^ AFM scans showing increasing nanoparticle coverage: 1.0 g/L for 4 h (b), 2.0 g/L for 4 h (c), 4 g/L for 1 h (d), and 4 g/L for 4 h (e).

Week-long immersion tests performed at different pH values demonstrated that both flat and porous CAB–PDA–Ag films maintain their integrity and optical characteristics (Supporting Information, Figure S4), confirming adequate stability under the conditions relevant to this work.

The antibacterial activity of the functionalized surfaces against Gram-negative *E. coli* was evaluated by assessing bacterial viability after 24 h of contact. Results were compared to control cultures grown under identical experimental conditions in the absence of the test materials. We first evaluated the effect of CAB surfaces, both flat and microporous, without PDA functionalization (Figure 5). The flat CAB film showed no measurable antibacterial activity. In contrast, the surface morphology of the microporous CAB films significantly influenced antibacterial performance: both P2000 and P4000 reduced the viability of planktonic and sessile *E. coli* populations by more than 50% compared to that of untreated controls. The comparison of P2000 and P4000 did not show significative statistical difference both for planktonic and sessile forms.

**Figure 5:**
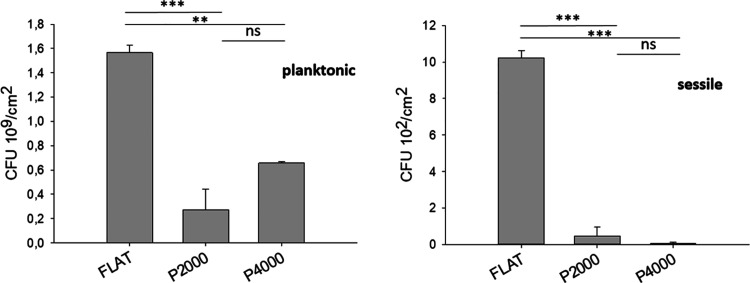
Viability tests of planktonic and sessile bacteria in the presence of different films. Data are presented as mean ± standard deviation. Differences between samples were evaluated using one-way analysis of variance (ANOVA) followed by Bonferroni post hoc testing. All the comparisons passed the ANOVA test. The Bonferroni *t* test results are reported in the figure as: ns (p > 0.05), * (p < 0.05), ** (p < 0.01), *** (p < 0.001).

To better interpret the antibacterial response of the pristine microporous CAB films, we compared their *E. coli* surface coverage with that of the flat CAB substrate using quantitative analysis of SEM micrographs (see Figure S2 in the Supporting Information). After 24 h of incubation and removal of the bacterial suspension, flat CAB displayed the highest density of adherent bacteria, followed by P2000 and then P4000. Notably, this ordering (flat > P2000 > P4000) mirrors the results of the sessile viability assay, which quantifies the fraction of bacteria that remain attached to the substrate and retain a colony-forming ability. Although the absolute numbers differ (SEM quantifies all adherent cells, both viable and nonviable, whereas CFU counts capture only surviving cells and involve more extensive rinsing), the consistent trend across the two independent measurements indicates that microporous CAB surfaces reduce bacterial adhesion rather than promote enhanced attachment. These findings support the interpretation that the decreased viability observed on P2000 and P4000 arises from an intrinsic inhibitory effect associated with the microporous architecture.

Next, we investigated the antibacterial effect of PDA by comparing *E. coli* cell viability on PDA-coated flat CAB surfaces versus uncoated CAB controls. As shown in Figure 6, the presence of PDA resulted in a limited reduction in the planktonic cell viability (about 30%). The effect was more pronounced for sessile cells. On the PDA-coated flat CAB samples, sessile CFU counts were at or below the calculated LOD (∼12 CFU/cm^2^) in all replicates, corresponding to the absence of detectable viable surface-attached cells in the CFU assay, whereas the uncoated CAB displayed ∼10^3^ CFU/cm^2^ under identical conditions.

**Figure 6:**
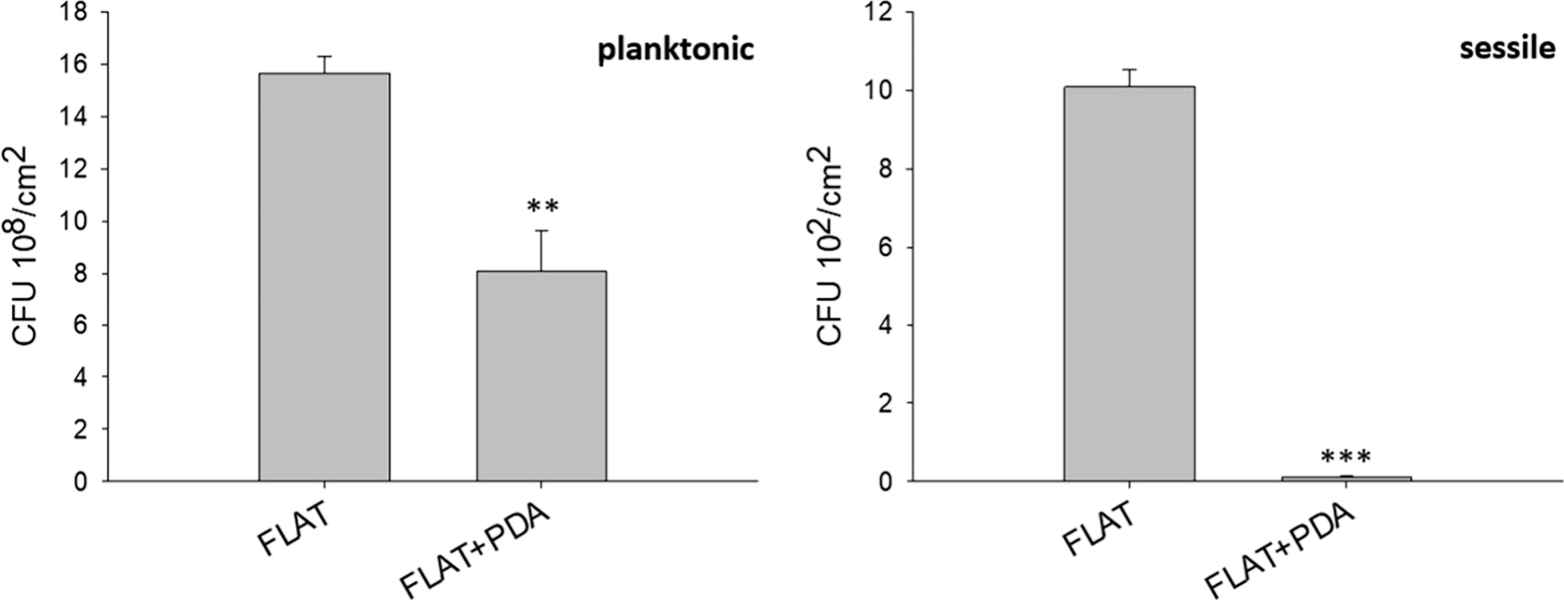
Effect of polydopamine. Data are presented as mean ± standard deviation. Statistical differences between samples were evaluated using a two-tailed Student’s *t* test: ns (p > 0.05), * (p < 0.05), ** (p < 0.01), *** (p < 0.001). Comparisons were performed between *E. coli* planktonic and sessile cells in the presence of coated vs uncoated films.

These outcomes are consistent with the physicochemical changes induced by PDA, particularly in surface hydrophilicity, as evidenced by the reduction of the water contact angle from 75 to 55°. Enhanced hydrophilicity modulates bacterial/surface interactions by altering protein adsorption and reducing the thermodynamic favorability of bacterial adhesion.^33^ In this context, the PDA-modified CAB impairs the initial attachment phase critical for biofilm development while also contributing to a less favorable environment for planktonic cells. Planktonic and sessile measurements probe distinct subpopulations governed by different mechanisms. Planktonic viability reflects effects occurring in the bulk medium (e.g., leached species and contact-independent stress), whereas sessile viability reflects the ability of bacteria to adhere and persist on the surface. Consequently, surfaces that suppress adhesion (e.g., PDA-modified CAB) can yield minimal sessile CFU but only partial decreases in planktonic CFU, as observed in Figure 6.

In the next set of experiments, presented in Figure 7, we evaluated the engineered surfaces combining both microporous structuring and the PDA coating. The antibacterial performance of these PDA-coated microporous materials was assessed by comparing the viability of *E. coli* across different substrate morphologies. A drastic reduction in planktonic cell viability (99.9%, corresponding to a 3-log decrease) was observed for both PDA-coated porous samples, confirming their strong antibacterial activity. The response of sessile cells, however, revealed a more complex behavior. Only a minor difference was observed between P2000 and its PDA-coated counterpart, whereas P4000 showed significantly lower biofilm viability compared to P2000. These differences appear to be primarily governed by the pore architecture rather than surface wettability. Both P2000 and P4000 exhibited highly uniform porous structures, with average pore diameters of approximately 640 and 470 nm, respectively. Although PDA coating reduced the WCA from ∼90° to moderately lower values, the surfaces remained within the hydrophobic range and cannot be considered truly hydrophilic. The finer pore structure of P4000 likely imposes greater spatial constraints on bacterial colonization, limiting the stability and expansion of sessile communities.

**Figure 7:**
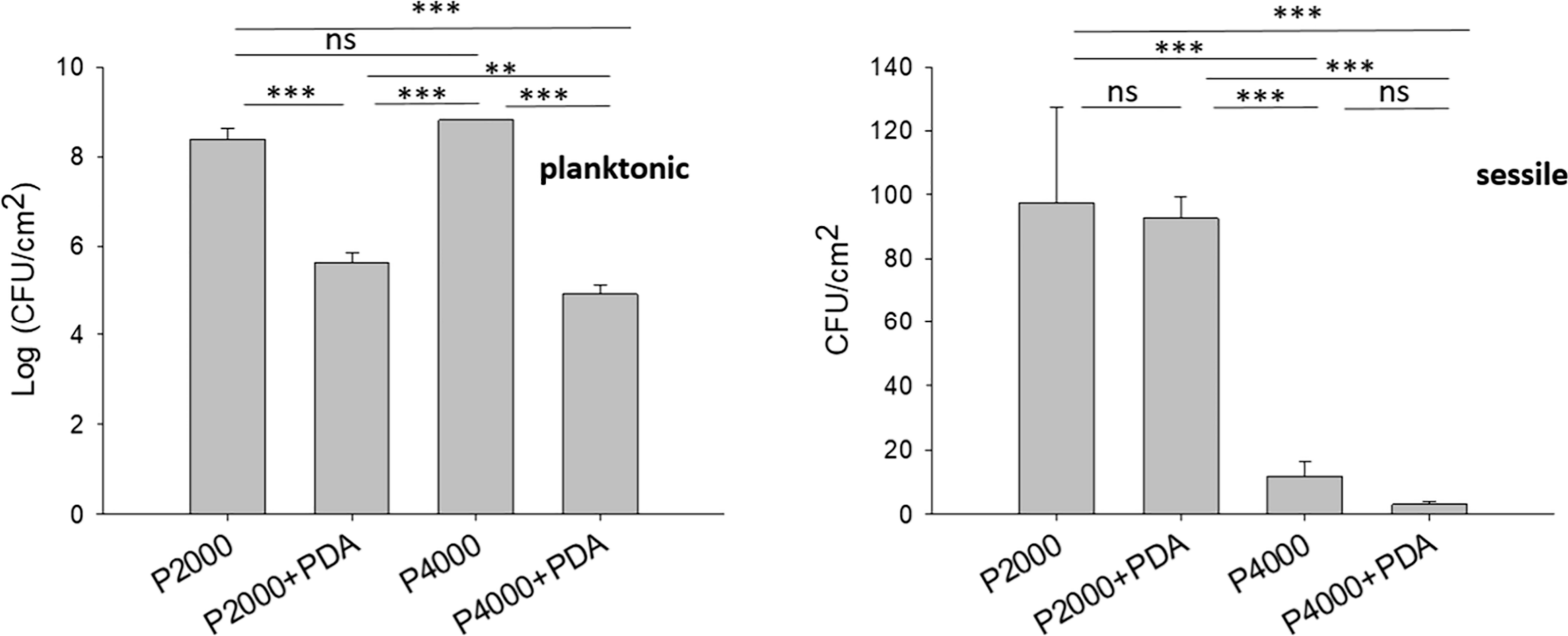
Combined effect of the morphology and polydopamine coating. Statistical differences between samples were evaluated using one-way ANOVA followed by Bonferroni post hoc testing. All the comparisons passed the ANOVA test. The Bonferroni *t* test results are reported in the figure as: ns (p > 0.05), * (p < 0.05), ** (p < 0.01), *** (p < 0.001). Comparisons were performed between *E. coli* planktonic and sessile cells in the presence of coated vs uncoated films.

SEM imaging after 24 h of incubation with *E. coli* reveals distinct bacterial colonization profiles across the investigated surfaces (Figure 8). The flat CAB sample, devoid of functional groups or topographical features, exhibits extensive bacterial adhesion and biofilm formation (Figure 8a), while the PDA-coated surface shows a lower bacterial load due to the presence of catechol and amine functionalities, which interfere with bacterial surface interactions (Figure 8b–d).

**Figure 8:**
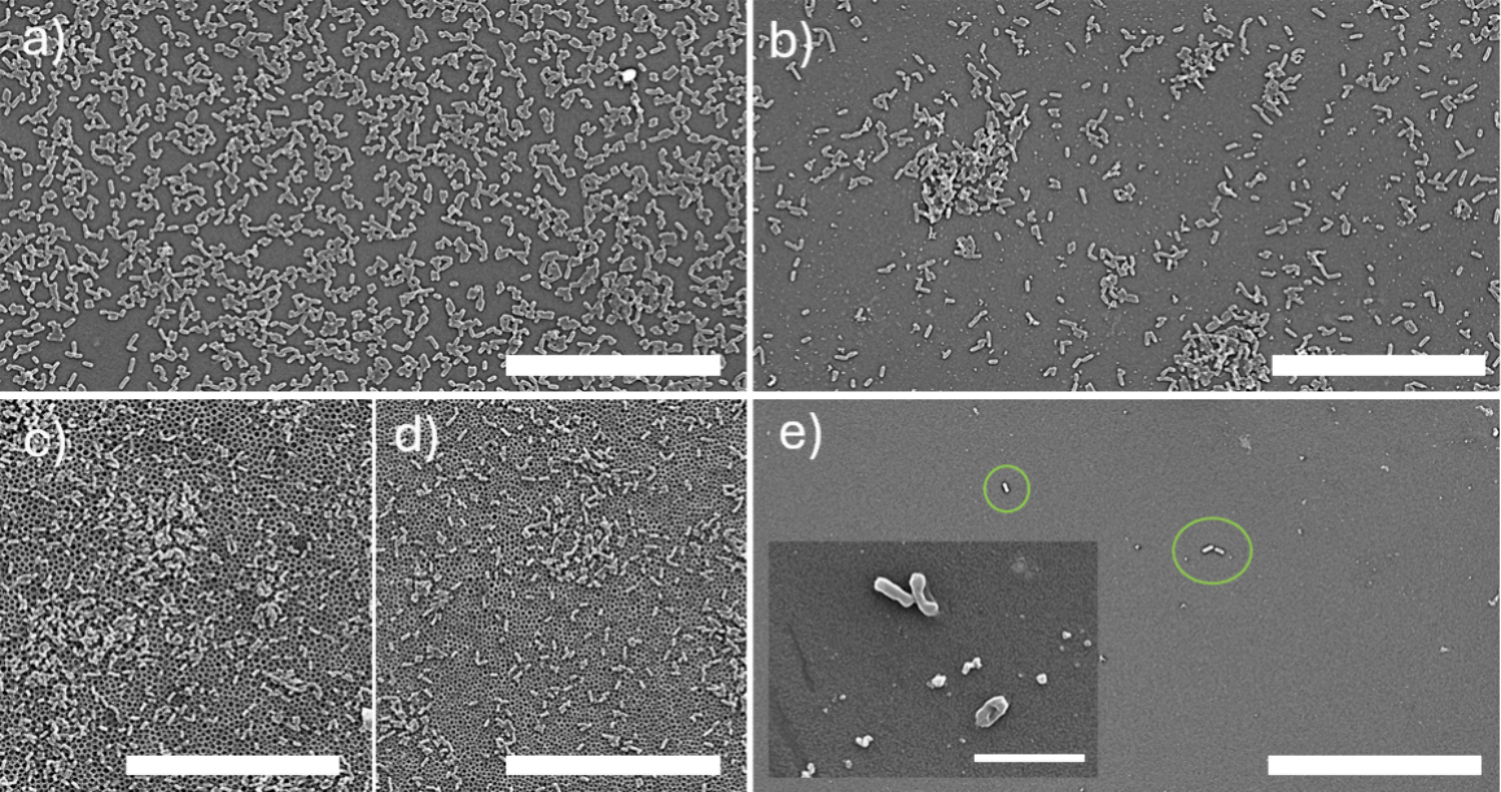
SEM view of different surfaces after 24 h of incubation with *E. coli*. Flat CAB (a), flat PDA (b), porous PDA 2000 rpm (c), porous PDA 4000 rpm (d), and flat PDA + AgNPs (e). Green circles in panel (e) highlight the bacterial cells. Scale bars are 10 μm (a–e) and 3 μm in the inset of panel (e).

A careful comparison of bacterial colonization in frames c and d of Figure 8 reveals that P4000, characterized by finer pores, appears to more effectively limit bacterial adhesion than P2000, which features larger pore structures, confirming the data of sessile colonization displayed in Figure 7.

The most pronounced antibacterial effect, as expected, is observed on the flat PDA-coated surface functionalized with silver nanoparticles (Figure 8e). This sample contains only a few isolated bacterial cells with no observable biofilm structures. In both planktonic and sessile assays, CFU counts were estimated below the LOD. The antibacterial inhibition observed for the AgNP-decorated surfaces is consistent with the release of Ag^+^ ions into the surrounding medium, as supported by the quantitative Ag^+^ release measurements reported in the Supporting Information. The synergistic action of PDA and AgNPs provides a dual antibacterial mechanism, disrupting both free-floating and surface-attached bacterial populations, resulting in the near-complete suppression of colonization.

A preliminary assessment performed on *S. aureus* did not reproduce the morphology-dependent antifouling trend observed for *E. coli*, and the results showed substantial variability that prevents drawing firm conclusions. The only consistent trend that we observed was a moderate inhibition on the AgNP-decorated samples. This lower sensitivity of *S. aureus* to Ag, compared with *E. coli*, is consistent with reports in the literature and is attributed to the thicker peptidoglycan layer and the distinct membrane charge characteristics of Gram-positive bacteria (see Figure S5 of the Supporting Information).^34^

Fluorescence microscopy performed after 24 h of incubation with *E. coli* and SYTO9 staining reveals bacterial distribution across the tested surfaces, in agreement with SEM observations. The flat CAB surface exhibits a strong fluorescence signal, consistent with extensive bacterial colonization (Figure 9a).

**Figure 9:**
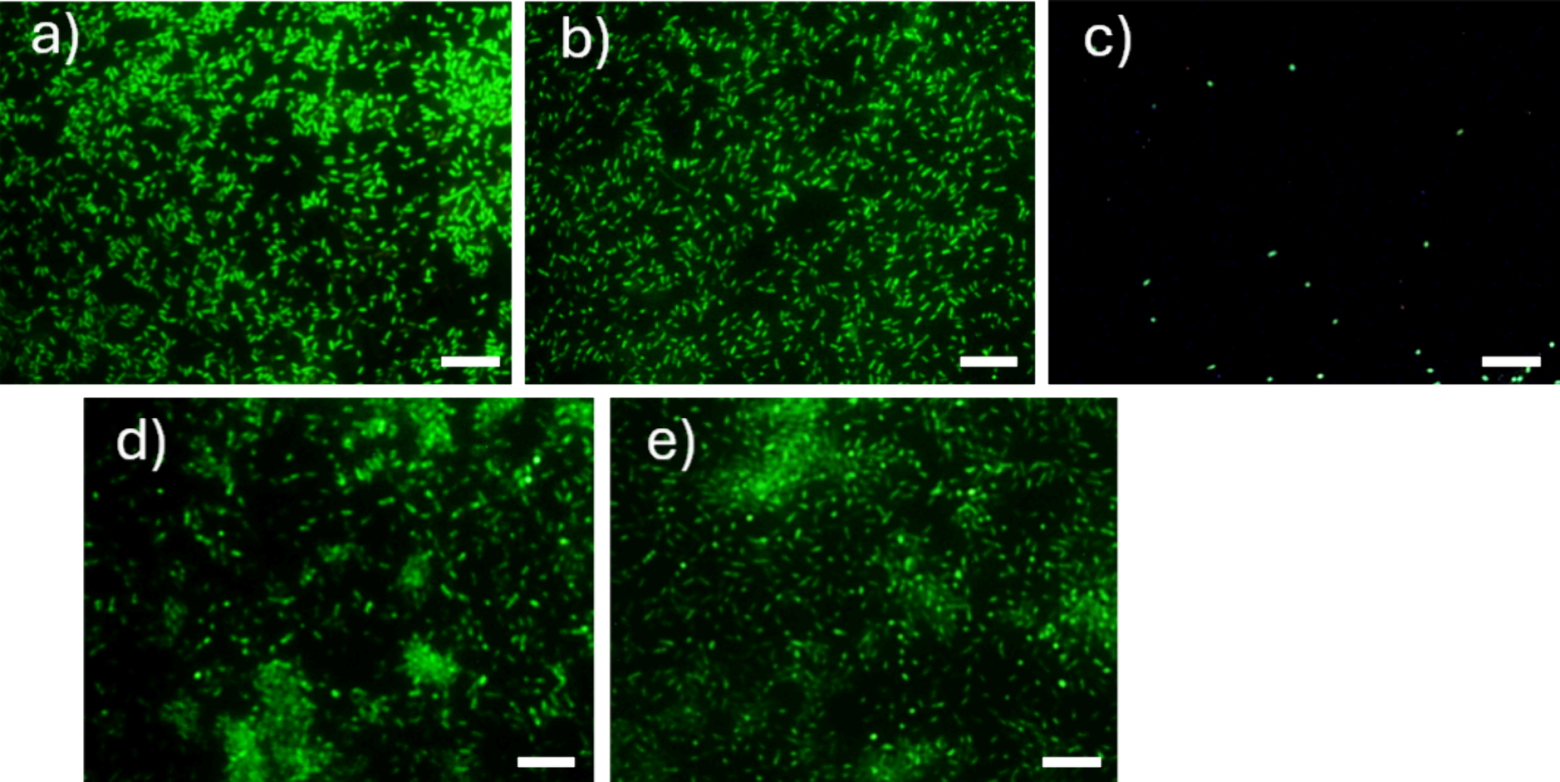
Fluorescence images of different surfaces after 24 h of incubation with *E. coli* and staining with green fluorescent SYTO9. Flat CAB (a), flat PDA (b), flat PDA + AgNPs (c), porous PDA 2000 rpm (d), and porous PDA 4000 rpm (e). Scale bars are 5 μm.

The flat PDA-coated surface exhibits a visibly reduced fluorescence signal compared to CAB, consistent with lower bacterial adhesion due to the presence of functional groups that interfere with cell–surface interactions (Figure 9b). The PDA surface functionalized with silver nanoparticles shows only a minimal bacterial presence, which confirms the potent bactericidal activity of AgNPs (Figure 9c). Signals from nonviable or transiently adhered cells can still be visible in imaging, even when CFUs are counted below the LOD.

The porous PDA-coated surfaces fabricated at 2000 and 4000 rpm exhibit comparable fluorescence signal intensities following SYTO9 staining (Figure 9d,e), despite the differences in bacterial colonization observed in SEM imaging. SEM analysis reveals that the finer pore architecture of P4000 disrupts stable adhesion and biofilm formation, whereas P2000 supports more extensive sessile colonization. The similarity in fluorescence signals reflects the presence of bacteria on both surfaces after incubation, but only SEM provides the spatial resolution necessary to distinguish between loosely associated cells and those forming structured biofilms.

## 4 Conclusions

In this study, we developed a sustainable strategy for fabricating multifunctional antibacterial surfaces by integrating microporous CAB, a bioinspired PDA coating, and in situ synthesized AgNPs. CAB enabled rapid microstructuring via the BF technique, while PDA provided both adhesion and reductive functionality for AgNP deposition without external agents. Antibacterial assays against *E. coli* demonstrated that bacterial viability is strongly influenced by both the physical architecture of the surface and its chemical functionality. The enhanced antimicrobial performance observed across the samples arises from a synergistic combination of tailored surface topography, bioinspired chemical modification, and silver-mediated bactericidal action. The overall fabrication process is characterized by low-energy input and minimal solvent usage, reflecting several principles of more sustainable materials engineering while simultaneously offering a versatile platform for the development of next-generation antibacterial coatings tailored for biomedical, environmental, and industrial applications.

## Supplementary Material



## Notes

Additional data figures and tables: AFM roughness analysis, evaluation of bacterial coverage on flat and porous films, quantification of silver release, stability tests, and viability of *S. aureus* (PDF)
