# Supplementary material for: Combining Topography and Chemistry toward Polydopamine Antibacterial Surfaces
Source: ACS Bio Med Chem Au. 2026 Apr 19;6(3):254–62. doi: 10.1021/acsbiomedchemau.5c00242 (PMC13281023; doi:10.1021/acsbiomedchemau.5c00242)
Supplement: Supplementary file 1 [file bg5c00242_si_001.pdf]

Supporting Information

Combining topography and chemistry towards polydopamine antibacterial surfaces

Leonardo Moscolari, Simona Tomaselli, Francesco Galeotti *, Erika Kozma *

Istituto di Scienze e Tecnologie Chimiche "G. Natta" (SCITEC), Consiglio Nazionale delle Ricerche, via A. Corti 12, 20133 Milano, Italy.

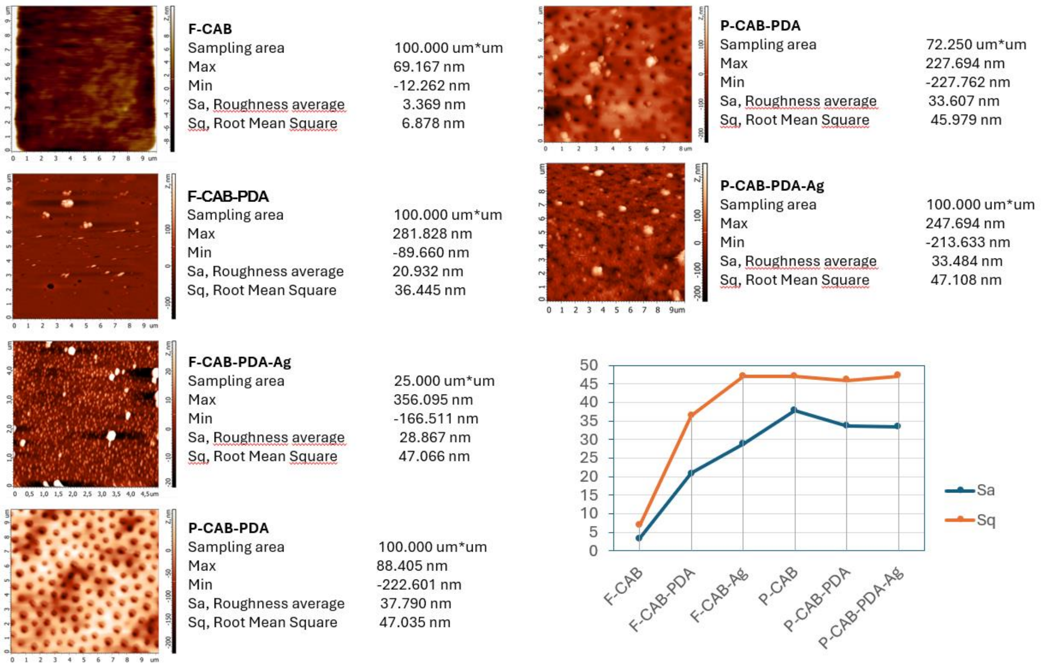

Figure S1. AFM roughness analysis of flat (F) and porous (P) CAB pristine films, after PDA coating and after Ag NP growth. Data of average roughness (Sa) and root mean square (Sq) are summarized in the plot. The roughness of porous samples is underestimated due to tip–sample convolution and limited probe accessibility in high-aspect-ratio porous morphologies.

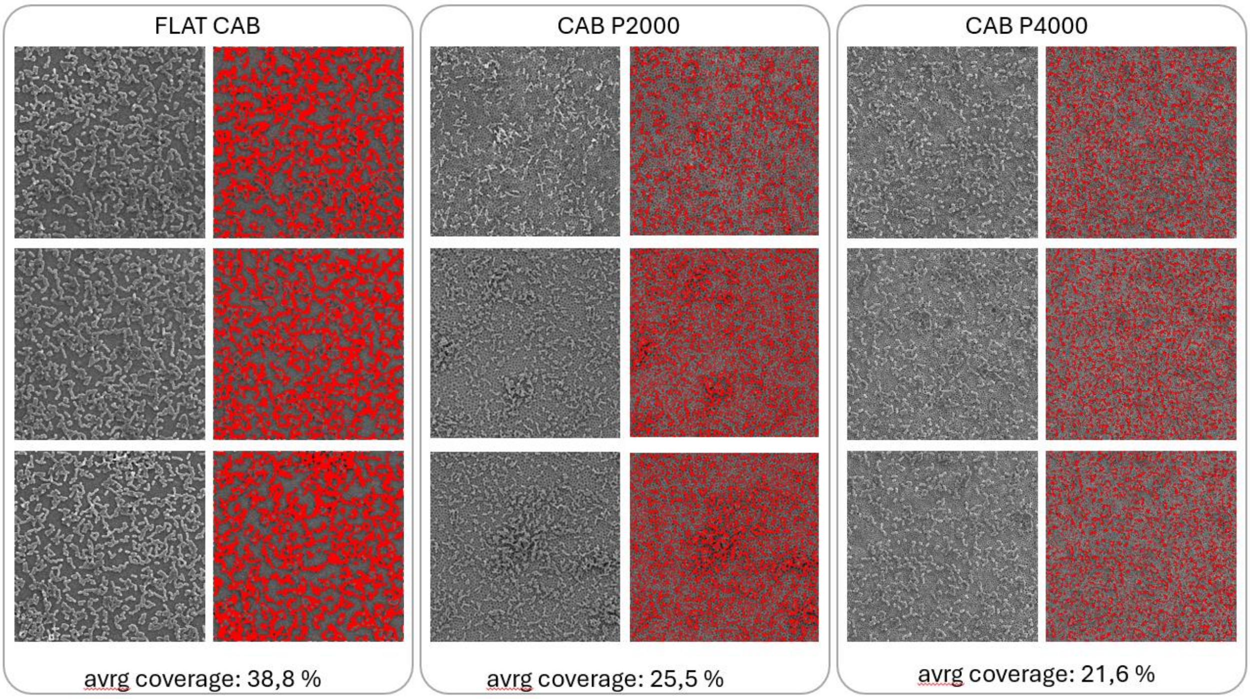

Figure S2. Evaluation of bacterial coverage on FLAT CAB, CAB P2000, and CAB P4000 surfaces after 24h incubation with pre-inoculated minimal medium followed by removal of the bacterial suspension. For each sample, the left panels show the original SEM images, while the right panels display the corresponding bacterial coverage masks obtained through adaptive local thresholding combined with morphological filtering to selectively segment bacterial cells while excluding micropores. The average surface coverage, calculated from three independent 50x50 μm^2 fields of views, is reported below each group.

Quantification of Ag^{+} release via photoluminescence quenching.

The photoluminescence (PL) intensity of core-shell CdSe/CdS quantum dots was measured in the presence of known concentrations of Ag^{+} within the expected range (5×10^{-5}–1×10^{-3} M) [1]. The corresponding PL spectra used to obtain the calibration curve is shown in Figure S3.

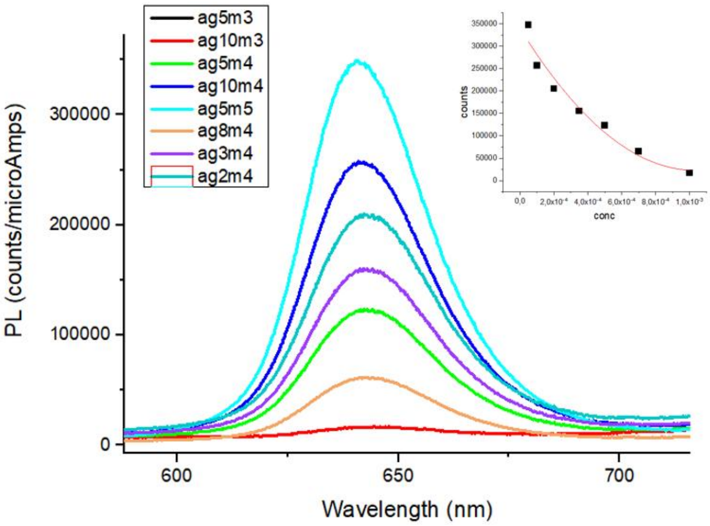

Figure S3. PL spectra of CdSe/CdS quantum dots aqueous solutions added with Ag^{+} (quenching metal ion) at different concentrations, from 5×10^{-5} to 1×10^{-3} M and corresponding calibration curve in the inset.

The calibration curve, obtained by plotting the Ag^{+} conc. Vs PL_{max}, was fitted using an exponential decay function of the form:

$$PL(C) = PL_0\, e^{-kC}$$

where $PL(C)$is the measured PL intensity at Ag^{+} concentration C, $PL_0$is the PL value at zero quencher concentration, and $k$is the quenching constant. Fitting the experimental calibration points yielded:

$$PL(C) = 4.01 \times 10^{5}\, e^{-3000\,C}$$

This equation was used to determine the Ag^{+} concentration in unknown samples by inversion of the exponential model:

$$C = -\frac{1}{k} \ln\left(\frac{PL}{PL_0}\right)$$

Applying this expression to the measured PL intensity of the sample after 24h of immersion in water at pH=7 (2900000 counts) gave:

C = 1.080×10^{-4} M

This concentration was converted to µg/mL of Ag^{+} using the molar mass of silver (MM(Ag^{+}) = 107.868 g mol^{-1}), resulting in 11.65 µg/mL (18.36 µg/mL referring to $AgNO_3$).

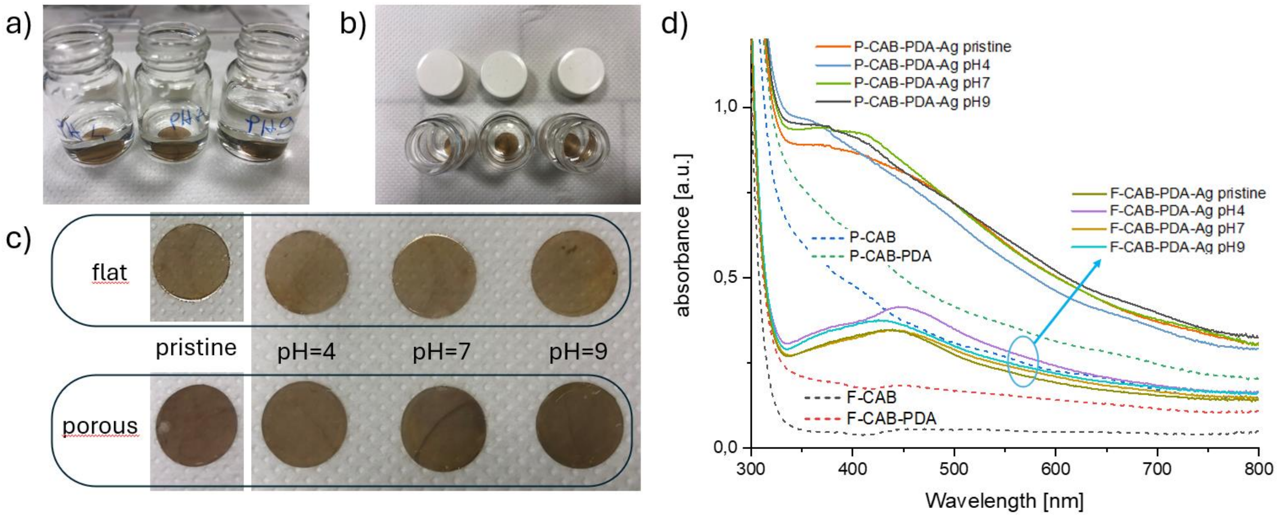

Figure S4. a-b) Photographs of stability test at different pH. c) Visual evaluation of the integrity of flat (top line) and porous (bottom line) CAB-PDA-Ag surfaces before (first left column) and after one week of incubation at different pH. d) UV-vis absorption plot of all samples pre- and post-incubation. Spectra of samples without AgNPs (F-CAB, F-CAB-PDA, P-CAB and P-CAB-PDA, untreated) are also shown as dashed lines, for reference.

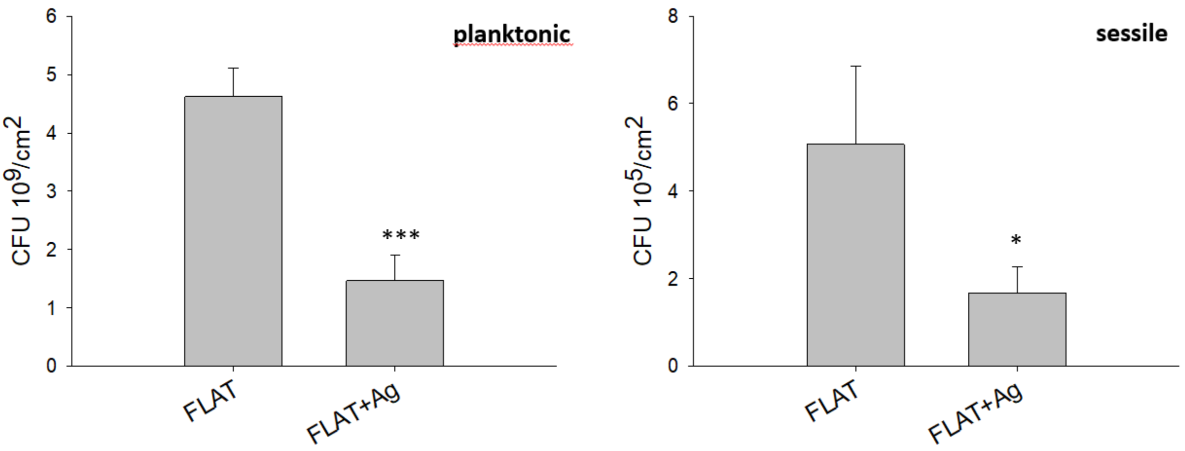

Figure S5. Viability tests of planktonic and sessile *S. aureus* bacteria in the presence of pristine flat CAB surface and of flat surface after PDA coating and decoration with Ag NPs. Statistical differences between samples were evaluated using a two-tailed Student's t-test; ns, $p > 0.05$; **, $p < 0.01$; ***, $p < 0.001$.

STRAIN	MIC	Reference
CMCC(B) 44102	10.2μg/mL (0.06mM $AgNO_3$)	[2]
ATCC 25922	8μg/mL ($AgNO_3$)	[3]
MG1655 (+IS1)	2.1μg/mL ($AgNO_3$)	[3]
hypermotile	1.7μg/mL ($AgNO_3$)	[4]
K12	4μg/mL ($AgNO_3$)	[5]
ATCC 8739	0.5μg/mL ($AgNO_3$)	[6]
ATCC 11229 (used in our study)	32μg/mL ($AgNO_3$)	[7]

Table S1. Data of Ag+ minimal inhibitory concentration (MIC) for *E. coli* reported in the literature.

REFERENCES

1. S. Wang, J. Yu, P. Zhao, S. Guo, and S. Han (2021) "One-Step Synthesis of Water-Soluble CdS Quantum Dots for Silver-Ion Detection." ACS Omega 6 (10), 7139-7146. DOI: 10.1021/acsomega.1c00162.
2. M.Tang, H.Yu, J.Deng, et al. "Enhancing the Antibacterial Effect of Silver Ions by a Cholesterol -2'-deoxycytidine Conjugate Against Escherichia coli and Staphylococcus aureus." ChemistrySelect11, no. 1 (2026): e01427. https://doi.org/10.1002/slct.202501427
3. Gimenez-Ingalaturre, A. C., Abad-Álvaro, I., Chueca, P., Goñi, P., & Laborda, F. (2025). Synergistic activity of silver nanoparticles and antibiotics: apramycin against Escherichia coli. Nanoscale Advances, 7(19), 6120–6131. https://doi.org/10.1039/d5na00404g
4. Stabryla, L. M., Johnston, K. A., Diemler, N. A., Cooper, V. S., Millstone, J. E., Haig, S.-J., & Gilbertson, L. M. (2021). Role of bacterial motility in differential resistance mechanisms of silver nanoparticles and silver ions. Nature Nanotechnology, 16(9), 996–1003. https://doi.org/10.1038/s41565-021-00929-w
5. Humphrey, B., Thomson, R., & Thomas, C. M. (2021). Role of bacterial motility in horizontal gene transfer and the spread of antibiotic resistance. PLOS Biology, 19(2), e3000292. https://doi.org/10.1371/journal.pbio.3000292
6. Li, W.-R., Sun, T.-L., Zhou, S.-L., Ma, Y.-K., Shi, Q.-S., Xie, X.-B., & Huang, X.-M. (2017). A comparative analysis of antibacterial activity, dynamics, and effects of silver ions and silver nanoparticles against four bacterial strains. International Biodeterioration & Biodegradation, 123, 304–310. https://doi.org/10.1016/j.ibiod.2017.07.015
7. Schierholz, J. M., Beuth, J., & Pulverer, G. (1999). Letter to the editor. Antimicrobial Agents and Chemotherapy, 43(11), 2819–2821.
